# Time-Dependent Negative Effects of Verbal and Non-verbal Suggestions in Surgical Patients—A Study on Arm Muscle Strength

**DOI:** 10.3389/fpsyg.2020.01693

**Published:** 2020-07-28

**Authors:** Nina Zech, Matthias Schrödinger, Milena Seemann, Florian Zeman, Timo F. Seyfried, Ernil Hansen

**Affiliations:** ^1^Department of Anesthesiology, University Hospital Regensburg, Regensburg, Germany; ^2^Department of Internal Medicine, District Hospital Wörth an der Donau, Wörth am Rhein, Germany; ^3^Department of Anesthesiology, Agaplesion Diakonieklinikum Hamburg, Hamburg, Germany; ^4^Centre for Clinical Studies, University Hospital Regensburg, Regensburg, Germany

**Keywords:** nocebo effects, dynamometry, maximal muscle strength, therapeutic communication, suggestions, State-Trait Anxiety Inventory (STAI)

## Abstract

**Introduction:**

The medical environment is full of suggestions that affect patients and their healing. Most of them inadvertently are negative, thus evoking nocebo effects. Recently, we have reported on the effect of such verbal and non-verbal suggestions as well as alternative formulations on maximal muscular arm strength in healthy volunteers. In the present study, we tested the same suggestions in patients at two time points to evaluate nocebo effects in a clinical situation and the impact of the approaching surgery date.

**Methods:**

In 45 patients, maximal muscular strength during arm abduction was measured by dynamometry of the deltoid muscle group. One test was several days before and the second on the evening before surgery. Baseline values were compared to the performance after exposure to 18 verbal and non-verbal suggestions. The sequence of presumably negative and positive suggestions was randomized for each patient in order to avoid cumulation effects of immediate succession of two negatives. State anxiety was evaluated at both time points, and suggestibility was measured after surgery.

**Results:**

Strong and statistically significant weakening effects were observed with all presumed negative suggestions from daily clinical practice including words of encouragement (91.4% of baseline), evaluation of symptoms (89.0%), announcement of a medical intervention (82.8%), a negative memory (86.5%), expectation of an uncertain future (82.8%), and non-verbal signals (87.7–92.2%). In contrast, alternative formulations did not interfere with muscular performance in most cases. A more pronounced effect was observed in the test repeated closer to the date of surgery, accompanied by a 15% higher anxiety level. The increase in anxiety correlated slightly with stronger weakening effects of suggestions, as did suggestibility.

**Conclusions:**

Negative suggestions cause a decrease in arm muscle strength, i.e., a “weakening” of the patient. This effect is enhanced by an increase in anxiety as the time of treatment, like surgery, approaches. The reaction can be avoided by alternative formulations. These nocebo effects that are objectively measured and quantified by a decrease in arm muscle strength are more pronounced in patients, i.e., in a clinical situation, than in healthy volunteers.

## Introduction

In daily clinical practice, many situations and communications between doctor and patient are suspected to elicit nocebo effects. However, concrete evidence often is limited. One of the reasons for this discrepancy is specificity. For instance, pain is induced or amplified by words about pain, nausea is increased by asking a patient about nausea, and side effects increased, if they were addressed (Lang et al., 2005; Varelmann et al., 2010; Häuser et al., 2012). “Words hurt” describes the observation that pain-related words affect pain (Lang et al., 2005; Corsi et al., 2019). Other effects of the same words may be missed when only pain is evaluated. The demonstration of a nocebo-induced symptom is largely dependent on the symptoms and physiological parameters in focus. These are limited and limiting. Many presumed effects on patient’s health, such as on the immune system or on wound healing, are difficult to define and to measure, and immediate changes may not be observable in a timely manner (Wobst, 2007). The longer it takes to get the result of the intervention, the higher the uncertainty in the assessment of its outcome. Moreover, specificity of the nocebo effects hampers comparisons. Is a nocebo effect on nausea stronger or weaker than on pain or on sexual dysfunction?

We recently presented a different approach to studying nocebo effects by measuring changes in maximal arm muscle strength as a general parameter for a “weakening” and as an immediate reaction to a nocebo induction by verbal and non-verbal suggestions (Zech et al., 2019). In a study on healthy volunteers, we demonstrated significant impairment in this one uniform objective physiological parameter after exposure to different suggestions, both verbal and non-verbal, common in routine clinical practice. Each challenge was compared to an alternative wording or visual presentation demonstrating that the nocebo effects can be avoided. Therefore, this technique allows for improvements in medical communication guided by objective measures.

Here we present the results of a subsequent study testing the same clinically relevant suggestions in patients at two time points prior to surgery. We hypothesized that the effects would be more pronounced in the clinical situation, i.e., in patients as compared to healthy volunteers, and that the effects increase as the time of surgery is coming closer.

## Materials and Methods

### Design and Participants

This experimental, randomized study was conducted at the University Hospital Regensburg, Germany, after approval by the local ethics committee (EC University of Regensburg, Nr. 13-101-0030). Patients were considered eligible for enrollment if they were between 18 and 70 years of age and were to undergo elective surgery under general anesthesia at the Departments of General Surgery, Neurosurgery, Otorhinolaryngology, or Cranio- Maxillofacial Surgery. Participants had to be native German speakers and with their surgery scheduled no closer than 3 days. Patients with pain [Numeric Rating Scale (NRS) > 5] and patients with pain or impairment at the dominant shoulder, arm, or hand were excluded. Another exclusion criterion was a preexisting severe systemic disease, as classified according to the ASA physical status classification system of the American Society of Anesthesiologists by a score of 3 or more. Fifty patients fulfilling the inclusion criteria were enrolled after written informed consent and without financial compensation. The patient information for the study included the request to abstain from coffee, Coke, or medication for the last 4 h before the test to avoid interference with motor performance.

### Measurement of Maximal Muscle Strength Under Suggestion

Maximal muscle strength under suggestions was measured at two time points: days before surgery (T1, minimum 3 days, median at day 3, 53% at day 3, the rest distributed around day 6 before surgery) and in the evening before surgery (T2). Maximal isometric contraction of the deltoid muscle group was tested by dynamometry in a defined upright position with the dominant arm stretched out laterally, as described previously (Zech et al., 2019). A dynamometer (FORCE GAUGE FM200, PCE Deutschland GmbH, Meschede, Germany) with a capacity of 196.0 N and a resolution of 0.05 N was used in the peak hold mode. Results were expressed as a percentage of the baseline value that was determined in 9–11 measurements for each subject. These relative values were used to respect the high variance of muscle strength between individuals. Maximal muscle strength measured under these conditions is a rather robust physiological parameter with an expected variation of ±6.3% from baseline (Zech et al., 2019). All patients were tested by the same examiner (M.Sch.). Each test session lasted about 40–60 min, which was found feasible even for patients and limited the number of tested suggestions to 18.

### Tested Suggestions and Application

The same suggestions out of clinical context were tested as in a previous study on healthy volunteers (Zech et al., 2019). Patients listened to recorded instructions explaining the placement and functionality of the muscle test, whereas suggestions were given verbally, face to face. Visual suggestions, including pictures or video clips, were projected on a notebook. Baseline was established by means of six initial measurements without suggestion followed by 3–5 such baseline measurements interspersed between tests of suggestions, adding up to a total of 9–11. The wording of the instructions prior to suggestions, as well as the type of suggestion itself, can be seen in Tables 1, 2. Nine clinical situations were evaluated. Version A of each suggestion was taken from everyday clinical practice and presumed to be negative and causing a nocebo effect. For each situation, an alternative version B was formulated, considered to be positive and to elicit a neutral or placebo effect. After six baseline measurements, suggestions were tested in a randomized order using the software Randlist (Datinf GmbH, Tübingen). In every patient, any presumed negative version was followed by a presumed neutral or positive version to avoid cumulation effects. Tests were separated by breaks, arithmetical tasks, and repeated determinations of blank values. To prevent incorrect measurements because of exhaustion, an additional break was inserted, whenever a baseline value fell below 90% of the previous, and the test was repeated subsequently.

**TABLE 1:** Wording of the standardized instructions and verbal suggestions.

Category	Scenario	Instructions	Version A	Version B
Baseline		“Now pull upward with maximal power. Now, one–two–three.”		
Sentences	Encouragement		You don’t need to be afraid. Don’t worry.	We are right by your side until you have successfully finished your procedure.
	Checking symptoms	“Again, stand upright, lift your arm. Close your eyes. You are a patient in a hospital. You are faced with the following sentences. Take your time and let it affect you, and then pull upward as hard as you can.”	Let us know when you feel pain. Do you feel nauseous?	Let us know if there is anything to make you feel better. We always can do something good for you. Do you feel OK?
	Doctor’s introduction and induction of anesthesia		Hallo, I’m Dr. Smith. I’ll put you to sleep now. We’ll start with the first drug, which will make you feel drowsy or drunk. Now we’ll start the second drug, which will burn a little bit. It will be all over soon.	Hallo, I’m Dr. Smith, your anesthetist. I’m here for your comfort and your safety. We are starting with a strong analgesic now that will make everything easier. Now I am giving you the second medication that will induce a restful sleep. I will be right by your side until you have finished your procedure successfully.
	Risk information for informed consent		If you wish, we can place a pain catheter, with the risk of infection, allergic reaction, and damage to blood vessels or nerves.	We have the option of a catheter to prevent discomfort. Even though there is a risk of infection, allergic reaction, or damage to blood vessels or nerves, you will have to take fewer pills, are more mobile, feel and recover better, and perhaps can go home sooner.
Situations	Conditioning	“Again, stand upright, lift your arm. Close your eyes and imagine the situation I suggest to you. When you are there, please nod and then pull upward as hard as you can.”	Negative memory: remember a situation, where something went really wrong. Everybody was disappointed in you, including yourself. It was terrible. You were really ashamed.	Positive memory: remember a situation when you were really successful and entirely satisfied with yourself. Everything went so well—totally perfect.
	Condition		Uncertain future: imagine an uncomfortable situation is about to take place: an impending operation, a performance review with your boss, an exam, or a confrontation with your partner. The result is uncertain.	Presence: you are fully in the here and now. You can feel the solid ground under your feet, notice your breath and your upright position while your mind is clear and open.

*For dynamometry of maximal arm muscle strength (arm abduction), the patient is standing upright facing the tester, with the right arm stretched to the side and the wrist connected to the dynamometer by a band.*

**TABLE 2:**
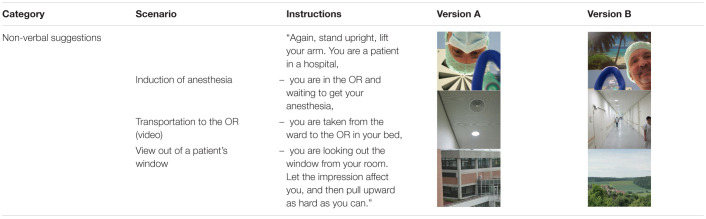
Wording of the standardized instructions and pictures of the non-verbal suggestions.

*For dynamometry of maximal arm muscle strength (arm abduction), the patient is standing upright with the right arm stretched to the side and the wrist connected to the dynamometer by a band, with pictures and video clips projected in front. (All six pictures were taken by one of the authors, EH; the upper two pictures show one of the authors, EH; the persons visible in picture “transportation, version B” gave permission).*

Possibly accepting a lower clarity, we deliberately refrain from designating the tested suggestions as “placebo” or “nocebo” in order to recognize the fact that we tested actual clinical situations, with “nocebo effect” as possible result, not as the test object.

### Measurement of Suggestibility

To explore the patients’ suggestibility, a five-item short version of the HGSHS (Riegel et al., 2020) was used. The HGSHS is an objective test method by Shor and Orne from 1962 to determine the suggestibility of a person or groups (Shor, 1962; Bongartz, 1985; Peter et al., 2015). The short version lasts about 25 min. Patients conducted it with an audio file a few days after their operation. Self-evaluation results in a maximum score of 5. Based on the HGSHS-5 score, patients were rated “low suggestible” (LS) with a score of 0 or 1, “medium suggestible” with a score of 2 or 3, and “high suggestible” (HS) with a score of 4 or 5.

### Measurement of Anxiety

Anxiety was measured with the state scale of the STAI-S with 20 test items in a German version (Laux et al., 1981). Evaluation took place at the two mentioned time points to draw conclusions about variations of anxiety over time with the approaching operation date. With a range of 20 (“no fear”) to 80 (“worst fear”) points, the test evaluates the current situational anxiety. Anxiety is usually considered clinically relevant at a score >40, and at >55 rated relevant for psychiatric disorders (Knight et al., 1983; Addolorato et al., 1999). The difference between the scores at T2 and T1 is referred to as ΔSTAI-S and describes the change of anxiety between the two different points in time.

### Statistical Analysis

Normal distribution of results was tested with the Kolmogorov–Smirnov test. With non-normal distribution, equality of force regarding baseline, version A and version B, was examined by Friedman two-way analyses of variance by ranks. For significantly different results, the Wilcoxon rank-sum test was used *post hoc* for pairwise testing. An α-error correction has been omitted to avoid the loss of possible correlations (Bender and Lange, 2001). Time-dependent (T1 vs. T2) differences of muscle strength were calculated using the Wilcoxon test, or rather Student’s *t*-test for anxiety level. Univariate linear regression analysis was performed for each suggestion. Multivariate linear regression analysis was performed for significant results to investigate influences of various parameters on muscle strength, i.e., gender, age, suggestibility, anxiety, and change in anxiety (ΔSTAI-S). For testing unconnected samples, e.g., the differences in gender or age groups, the Mann–Whitney *U* test was used. Significance level was assumed as *p* < 0.05. Statistical analyses were performed with IBM SPSS Statistics, version 23.

## Results

### Baseline Characteristics

Out of 50 recruited patients, five were excluded because of missing data (only tested at T1, because patient declined, surgery rescheduled or canceled). Characteristics and baseline scores of the remaining 45 are presented in Table 3. Due to the individual physical condition of the patients, baseline muscle strength ranged from 18.8 to 143.7 N. The reproducibility of the neutral values of each individual patient was high (variance 4.80% at T1 and 4.67% at T2). Baseline values did not differ significantly at T1 and T2 (*n* = 0.871). For further analysis, patients were stratified in “younger” (<45 years, *N* = 19) and “older” (≥45 years, *N* = 26) according to the median. Suggestibility was not normally distributed, with 12 patients (27%) scoring LS and 10 patients (22%) HS.

**TABLE 3:** Baseline characteristics of study population (*N* = 45).

Age (years)	Mean ± *SD*	43.8 ± 15.0
Female sex	*N* (%)	25 (56%)
Suggestibility (HGSHS-5)	Median (IQR)	3 (1–3)
Anxiety (STAI-S)	Mean ± *SD*	41.7 ± 10.3
Days from first test to surgery	Mean ± *SD* (range)	5.7 ± 4.8 (3–25)
Baseline muscle strength (Newton)	Mean ± *SD*	
Days before surgery (T1)		65.0 ± 23.4
Evening before surgery (T2)		64.8 ± 23.5

*HGSHS, Harvard Group Scale of Hypnotic Susceptibility; STAI-S, State-Trait Anxiety Inventory; IQR, interquartile range. Baseline muscle strength did not differ significantly at T1 and at T2 (p = 0.871).*

### Time Course of Anxiety

Anxiety (STAI-S) raised significantly from a mean of 41.7 ± 10.3 to 47.9 ± 12.7 the night before the operation, with mean ΔSTAI-S of 6.2 ± 8.9 (*p* < 0.001). Neither age nor gender affected the level of state anxiety at T1 and T2; however, both had an impact on the increase in anxiety. In linear regression analyses, age had a significant effect on ΔSTAI-S, with younger patients showing a higher increase in anxiety (*R* = −0.385; *p* = 0.012). ΔSTAI-S was significantly higher in women (9.4 ± 9.2; *p* = 0.009). In multivariate regression analyses, age and sex were responsible for 31.3% of variance of ΔSTAI-S (*R* = 0.560; *p* = 0.001). Suggestibility had no significant influence on anxiety or anxiety increase. With a ΔSTAI-S of 24–27 points, three patients experienced a particularly strong reaction. Number of patients having a score >55 increased from five at T1 to 13 at T2. Out of these eight patients, seven were women, and seven were younger than 45 years.

### Effects of Sentences

**FIGURE 1:**
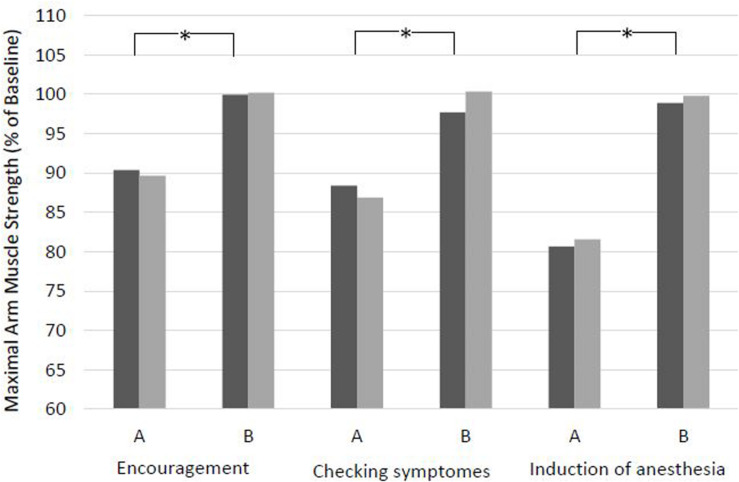
Effects of sentences with clinical context in two versions on maximal arm muscle strength. After baseline dynamometry of arm abduction, verbal suggestions were presented and measurement was repeated. T1, days before surgery; T2, evening before surgery. Mean of maximal arm muscle strength compared to baseline is given. *p < 0.01 (Wilcoxon rank sum test) at T1 and at T2.

Every version A of a sentence within the clinical context presented to the patients resulted in a highly significant reduction in maximal arm muscle strength at both time points, by 8.6–17.2% at T2 (Figure 1 and Table 4). The presumably negative words of a doctor to introduce himself or herself before narcotic induction showed the greatest effects. Here, 10 patients showed a weakening to below 70% of baseline, with a lowest value of 36%. In contrast, every alternative version B was neutral in effect and did not weaken the patients and did not cause a significant attenuation compared to baseline. For both time points T1 and T2, the difference between versions A and B was highly significant for all tested suggestions. The greatest differences between versions A and B were for checking symptoms (40%) and narcotic induction (60%). For every phrase, neither version A nor version B resulted in a significant difference in muscle strength between T1 and T2. Similar effects were observed after risk information for informed consent in two versions. These data will be given in detail elsewhere.

**TABLE 4:** Effects of sentences within the clinical context on maximal arm muscle strength.

Suggestion	Version A, median (IQR)		Version B, median (IQR)	
	*p*		*p*	
	T1	T2	T1	T2
Encouragement	92.3 (84.8–97.7) *p* < 0.001	91.4 (84.9–95.0) *p* < 0.001	101.5 (95.0–106.3) *p* = 0.604	100.0 (96.9–103.4) *p* = 0.771
Checking symptoms	91.7 (79.7–96.4) *p* < 0.001	89.0 (82.9–94.4) *p* < 0.001	97.6 (94.1–103.7) *p* = 0.099	100.2 (96.4–104.6) *p* = 0.809
Doctor’s introduction and narcotic induction	83.7 (72.4–89.3) *p* < 0.001	82.8 (75.3–90.8) *p* < 0.001	97.8 (96.6–103.9) *p* = 0.264	99.2 (95.8–103.7) *p* = 0.578

*After baseline dynamometry of arm abduction, verbal suggestions were presented and measurement was repeated. Median and interquartile range (IQR) of relative values compared to baseline (in %) after suggestion. T1, days before surgery; T2, evening before surgery. p according to Wilcoxon rank sum test relates to the difference to baseline after significance in Friedman test.*

### Effects of Situations

**FIGURE 2:**
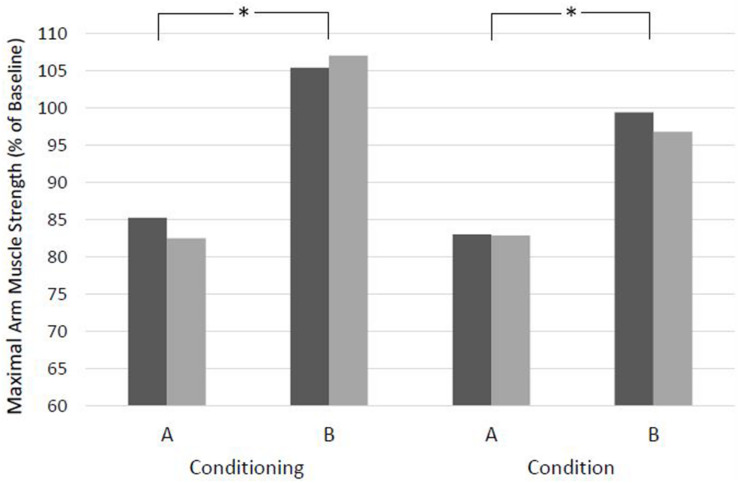
Effects of situations with clinical context in two versions on maximal arm muscle strength. After baseline dynamometry of arm abduction, verbal suggestions were presented and measurement was repeated. T1, days before surgery; T2, evening before surgery. Mean of maximal arm muscle strength compared to baseline is given. *p < 0.01 (Wilcoxon rank sum test) at T1 and T2.

Both the recall of a negative memory (Conditioning version A) and the idea of an uncertain, negative future (Condition version A) resulted in highly significant weakening at both time points (Figure 2 and Table 5). Ten and 12 patients, respectively, showed values under 70% of baseline, with minimum values of 49 and 58%. Suggestion of a positive, encouraging memory (Conditioning version B) was the only one resulting in a strengthening of the patients (T1 *p* = 0.008, T2 *p* < 0.001) compared to baseline. In 10 patients, version B of Conditioning raised muscle strength above 115% of baseline, with maximum values of 125%. The orientation to the presence (Condition version B) did not result in significant differences from baseline at any time point. For both situations, the difference between the two versions was highly significant for both times of measurement. Maximum difference was 55% for Conditioning and 45% for Condition. There was no significant difference between T1 and T2 for both versions of the two situations.

**TABLE 5:** Effects of situations on maximal arm muscle strength.

Suggestion	Version A, median (IQR)		Version B, median (IQR)	
	*p*		*p*	
	T1	T2	T1	T2
Conditioning	87.1 (80.1–93.7) *p* < 0.001	86.5 (75.6–90.8) *p* < 0.001	103.3 (97.4–113.8) *p* = 0.008	106.5 (100.9–114.8) *p* < 0.001
Condition	86.6 (75.7–92.4) *p* < 0.001	82.8 (74.0–88.9) *p* < 0.001	97.6 (92.7–107.0) *p* = 0.676	94.2 (90.8–104.1) *p* = 0.052

*After baseline dynamometry of arm abduction, verbal suggestions were presented and measurement as repeated. Median and interquartile range (IQR) of relative values compared to baseline (in %) after suggestion. T1, days before surgery; T2, evening before surgery. p according to Wilcoxon rank sum test relates to the difference to baseline after significance in Friedman test.*

### Effects of Non-verbal Suggestions

**FIGURE 3:**
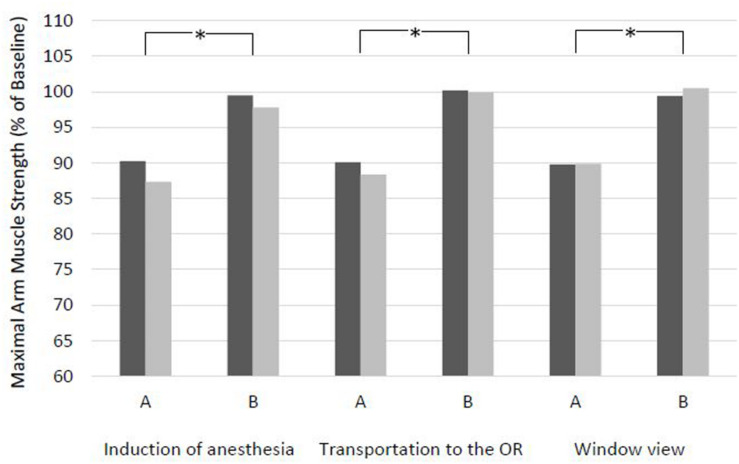
Effects of non-verbal suggestions with clinical context on maximal arm muscle strength. After baseline dynamometry of arm abduction, non-verbal suggestions were presented by projection and measurement was repeated. T1, days before surgery; T2, evening before surgery. Mean of maximal arm muscle strength compared to baseline is given. *p < 0.01 (Wilcoxon rank sum test) at T1 and at T2.

Every version A of a non-verbal suggestion presumed negative resulted in a reduced maximal arm muscle strength (88–92%), with a highly significant difference compared to baseline at both time points. Lowest values were 54, 67, and 54%, respectively (Figure 3 and Table 6). The alternative version B of the non-verbal suggestions was found to be neutral and did not result in a significant attenuation. The suggestion of a patient being transported to the OR in an upright position in his bed even strengthened the patients at T2 (*p* = 0.019), with a maximum score of 128%. The difference between versions A and B was highly significant for all non-verbal suggestions at both time points, with a maximum of 45% for Induction of anesthesia and View out of a patient’s window. There was no significant difference between T1 and T2 for any version of the three suggestions.

**TABLE 6:** Effects of pictures and video clips within the clinical context on maximal arm muscle strength.

Suggestion	Version A, median (IQR)		Version B, median (IQR)	
	*p*		*p*	
	T1	T2	T1	T2
Induction of anesthesia	89.9 (84.3–97.2) *p* < 0.001	87.7 (79.7–94.6) *p* < 0.001	101.8 (97.4–106.8) *p* = 0.194	99.8 (94.0–104.2) *p* = 0.984
Transportation to the OR	91.8 (84.1–97.2) *p* < 0.001	92.2 (80.7–96.2) *p* < 0.001	98.7 (93.8–106.2) *p* = 0.731	103.2 (97.6–109.7) *p* = 0.019
View out of a patient’s window	88.8 (82.5–93.9) *p* < 0.001	89.2 (82.0–95.3) *p* < 0.001	99.1 (95.3–105.4) *p* = 0.842	100.1 (97.6–105.9) *p* = 0.268

*After baseline dynamometry of arm abduction, non-verbal suggestions were presented by projection and measurement was repeated. Median and interquartile range (IQR) of relative values compared to baseline (in %) after suggestion. T1, days before surgery; T2, evening before surgery. p according to Wilcoxon rank sum test relates to the difference to baseline after significance in Friedman test.*

### Contributing Factors

**FIGURE 4:**
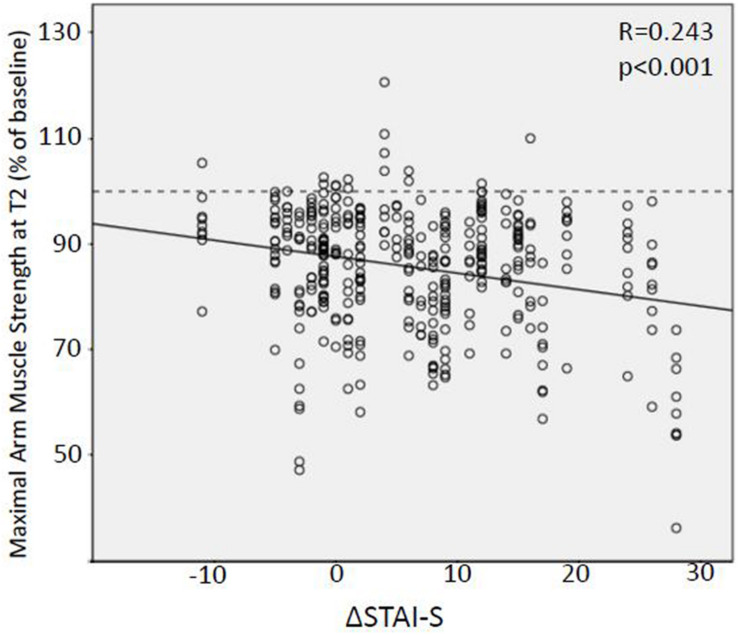
Linear regression analysis of the relation between preoperative anxiety increase and weakening effect of suggestions. Relative values of maximal arm muscle strength after version A of nine suggestions of clinical context tested on the evening before surgery (T2) plotted against the increase in state anxiety score (STAI-S) between several days before and at the evening of the surgery. STAI-S, State-Trait Anxiety Inventory.

Anxiety was found to have a marked influence on the effect of suggestions on maximal arm muscle strength. In linear regression analysis of all suggestions with significant weakening effects (respectively, versions A), STAI-S had no influence on the results at T1, whereas at T2, high anxiety scores led to enhanced weakening (*R* = −0.126; *p* = 0.012). Even more significant than anxiety itself was the effect of the increase in anxiety with the surgical date coming closer (ΔSTAI-S). In linear regression analysis, the reduction of muscle strength induced by suggestions both at T1 and T2 increased with higher ΔSTAI-S (T1: *R* = −0.212; *p* < 0.001; T2: *R* = 0.243; *p* < 0.001) (Figure 4).

**FIGURE 5:**
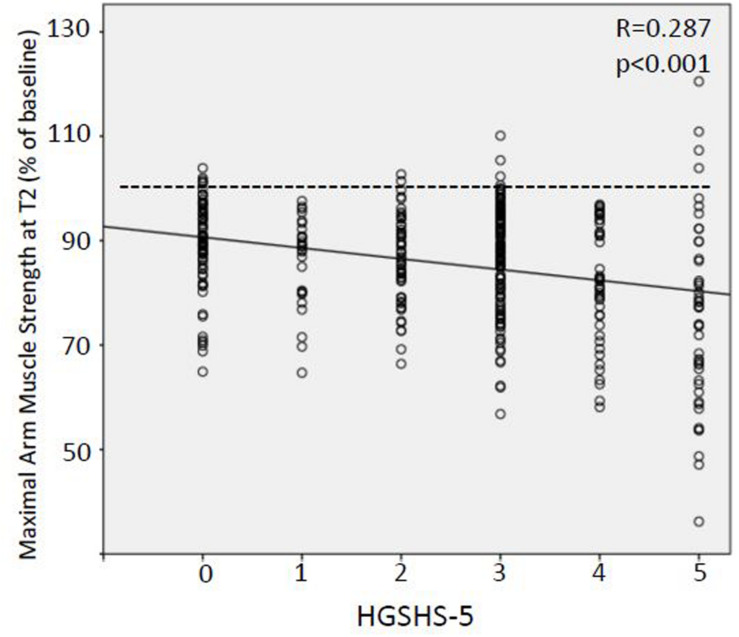
Linear regression analysis of the relation between suggestibility and weakening effect of suggestions. Relative values of maximal arm muscle strength after version A of nine suggestions of clinical context plotted against suggestibility score (HGSHS-5). HGSHS, Harvard Group Scale of Hypnotic Susceptibility.

Possible contribution of further factors was analyzed, namely, gender, age, and suggestibility. Linear regression analysis of all suggestions with significant effects showed impact of gender (*R* = 0.175, *p* < 0.001) and suggestibility (*R* = −0.172, *p* < 0.001) for T1. At T2, gender (*R* = 0.159, *p* = 0.002), age (*R* = 0.135, *p* = 0.008), and suggestibility (*R* = −0.287, *p* < 0.001) (Figure 5) had slight but significant effects. Especially in younger patients, women and patients with high HGSHS-5 score in negative suggestions resulted in pronounced weakening. In multivariate regression analysis, ΔSTAI-S and HGSHS-5 score were responsible for 12% of arm muscle strength’s variance at T2 (*R* = −0.345, *p* < 0.001).

## Discussion

Suggestions of clinical everyday routine turned out to elicit nocebo effects in patients by reducing maximal arm muscle strength in a time-dependent manner. Alternative formulations were able to avoid this weakening.

### Weakening Effect of Clinically Relevant Suggestions

Knowledge and awareness are increasing that the medical setting and the medical communication can exert negative effects on patients, their symptoms, and effectiveness of therapy (Häuser et al., 2012; Benedetti, 2013; Amanzio et al., 2020; Colloca and Barsky, 2020). Improved awareness, knowledge, and understanding of these nocebo effects can help to recognize triggers in the particular clinical field and work situation and thereby aim to avoid them (Hansen and Zech, 2019). For this purpose, it appears especially promising and appropriate to test relevant suggestions from daily clinical routine because they affect a high number of patients every day. In addition, what the patient usually experiences is not a single negative suggestion like in experimental settings, but a plurality of different inputs with complex interactions. Verbal and non-verbal signals interplay and are communicated all along during a hospital stay from admission to examination, from interview to risk assessment and information, from treatment to recovery. Moreover, in the clinical environment, the effects may add up to reach a complex aggregation of symptoms and impairments. While it is easy to demonstrate the effect of the words “pain,” “sting,” or “burn” on pain and the effect of the question “Do you feel sick?” or the sight of a bloody swab on nausea and vomiting, the combination may exert impairment of more general functions like comfort, anxiety, healing, or immune response (Lang et al., 2005; Wobst, 2007; Varelmann et al., 2010; Häuser et al., 2012). Most of such functions are complex and not easy or fast to measure, such as wound healing or immune surveillance. Moreover, they may be obscured in time by additional factors such as medication, hemodynamic instability, or complications. We therefore aimed to identify a common, albeit direct parameter to measure the immediate effects of different suggestions instead of direct connection between signal and symptom. With maximal arm muscle strength, we found a measurement fulfilling this criterion. In a study on healthy volunteers, we tested nine verbal or visual suggestions from everyday clinical practice in two versions and found a significant reduction in a performance that may be interpreted as marker for a “weakening” of the patient (Zech et al., 2019). Alternative formulations of these suggestions were able to neutralize the observed nocebo effect.

In the present study, testing the same paradigm and the same suggestions in the clinical situation on patients, again significant reductions in muscular function were observed at two different time points. Addressing a bad experience in the past tested a nocebo effect induced by conditioning, i.e., the patient’s own experience, and resulted in a significant weakening of arm muscle strength by 13.5% at T2 (Figure 2 and Table 5). This reflects the classical everyday situation of anamnesis that elicits a patient’s recall of prior disease and symptoms. Similarly, a condition projecting an uncertain and possibly negative future gives rise to a classical nocebo situation based on expectation. This resulted in the strongest weakening effect observed in this study (−17.2%). Encouraging words like “Don’t worry!” did not have the expected effect of a positive expectation and corresponding placebo effect, but instead resulted in significant weakening by 8.6% (Figure 1 and Table 4). An explanation may be the strong negative connotation of the word “worry” or “afraid” that cannot be neutralized by negation (Armstrong and Dienes, 2013; Hansen and Zech, 2019). Like in the proceeding study on volunteers, we also tested the effect of risk information for informed consent in this study and observed a reduction in muscle strength by 13.6% at T2 (detailed results will be published elsewhere to take into account the wide and special significance of this issue).

During the course of a hospital stay, the patient eventually undergoes a transport for medical treatment. In a strict supine position in his bed, he commonly experiences the view tested by a video clip (Table 2) with lamps and ventilation slots at the otherwise sobering blank ceiling. The observed reduction in muscle strength by about 8% by this non-verbal suggestion may appear small but adds to the many other negative influences (Figure 3 and Table 6). After arrival at the OR, the introduction of the doctor and the preparation of anesthetic induction are typical situations. Both the words and the overhead view of the anesthetist’s masked face (Table 2) induced significant impairment of strength, by 17.2 and 12.3%, respectively (Figure 1 and Table 2 or Figure 3 and Table 6). The image of the doctor’s face upside down, hidden behind a mask, interferes with biological face recognition (McKone et al., 2012). While tested separately, these verbal and non-verbal suggestions are experienced by the patient simultaneously in the clinical setting. After treatment, the patient is usually asked repeatedly about symptoms. The question about pain and nausea led to a weakening by 11% (Figure 1 and Table 4). Finally, the patient ends up at the ward where he might be confronted with a view on a parking lot or other dreary surrounding (Table 2). This visual perception induced a reduction in muscle strength by about 11% (Figure 3 and Table 6). Others have reported delayed recovery from surgery and increased consumption of analgesics (Ulrich, 1984). Our findings are in accordance with observation of non-verbal induction of placebo and nocebo effects (Daniali and Flaten, 2019). This course of a patient subsequently meeting different suggestions is a typical clinical situation in a hospital. In contrast to experimental studies in nocebo research, a patient is not exposed to a single challenge, but to multiple suggestions, possibly leading to summation effects. Therefore, we tested alternative formulations for suggestions all along this pathway of a patient through hospital stay.

The use of one common parameter to test the effects of different negative suggestions allows their direct comparison. The mean reduction in arm muscle strength by all nine negative suggestions tested was 14.4% compared to baseline (at T2). The uniform test parameter could also facilitate an evaluation of cumulative effects of different triggers that are simultaneously applied. Using this approach, further research may clarify whether concurrent nocebo effects are additive, attenuating, or potentiating.

### Alternative Formulations Avoid Nocebo Effects

The formulation of alternatives to the tested negative suggestions was successful in avoiding the nocebo effect. Weakening after exposure to version B was in a range of only 0 to −5.8%.

In some cases, even an increase in muscle strength was observed. This strengthening compared to baseline was significant, with a 6.5% increase after recall of a positive past, presumably by a classical conditioning reaction. Overall, for all nine suggestions, significance was not limited to the difference between baseline and version A, but also to the difference between the negative version (A) and the alternative (B). Therefore, version B evidently represents a better alternative for clinical practice. For instance, doctors should be aware of the weakening effect of asking about the medical history, which is inevitable, but could be neutralized by adding a question like “What was your preferred sport before your illness?” This utilizes the positive conditioned reaction to a positive recall tested with version B and should bring the patient out of the induced weakness. Similar effects are to be expected from shifting the focus to a positive future, “What are your plans after recovery from your surgery?” The effectively better alternative to the question “Do you feel nauseous?” is “Do you feel ok?” The non-verbal suggestions of the anesthetist face-to-face and a poster at the ceiling, the upright position during transportation in bed, and a view in the nature from the window actually are able to avoid the weakening effect of the original clinical situation. By the use of the uniform measurement parameter “maximal arm muscle strength,” not only can the alternative formulation be identified as qualitatively “neutral” or “not weakening” but also the effect can be quantified. Thereby, various alternatives could be tested and an optimal one found. Based on this method and principle, communication can be improved.

### Comparison to Results of Healthy Volunteers

Compared to the results of the preceding study on healthy volunteers, the effects of negative suggestions were more pronounced in patients (Table 7). The only exception was the transport in supine position that affected volunteers more than patients. A possible explanation for this difference is that counteractively to the terrifying view of the ceiling, the eagerly awaited treatment, namely, surgery, finally gets closer. The stronger reaction of the patients may be caused by the closer reality of the situation, especially on the evening before surgery. The reduction in muscle strength after the encouraging words “You don’t need to be afraid. Don’t worry.” was not only stronger, but reached significance. This draws attention to the possibility that many effects observed in experimental placebo/nocebo research might be much more pronounced in clinical situations.

**TABLE 7:** Weakening effect of clinically relevant suggestions in healthy volunteers and patients.

Suggestions	Volunteers	Patients T1	Patients T2
**Encouragement**			
A	−1.8	−7.7	−8.6
B	−1.5	+1.5	0
**Checking symptoms**			
A	−8.6	−8.3	−11.0
B	−2.4	−2.4	+0.2
**Doctor’s introduction and narcotic induction**			
A	−6.5	−16.3	−17.2
B	−0.6	−2.2	−0.8
**Risk information**			
A	−8.2	−12.6	−13.6
B	−3.6	−4.0	−1.2
**Conditioning**			
A	−10.6	−12.9	−13.5
B	+0.7	+3.3	+6.5
**Condition**			
A	−6.7	−13.4	−17.2
B	−4.6	−2.4	−5.8
**Induction of anesthesia**			
A	−9.0	−10.1	−12.3
B	−3.2	+1.8	−0.2
**Transportation to the OR**			
A	−10.7	−8.2	−7.8
B	−2.2	−1.3	+3.2
**View out of a patient’s window**			
A	−5.9	−11.2	−10.8
B	−3.4	−0.9	+0.1

*Relative difference to baseline of maximal arm muscle strength (in %) after verbal and non-verbal suggestions are given. Results of this study on patients at two different time points (T1, days before surgery; T2, evening before surgery) are compared to results of a preceding study on healthy volunteers (Zech et al., 2019).*

### Factors Contributing to Weakening Nocebo Effects

Although not reaching statistical significance, there was a trend to a more extended negative reaction the evening before surgery (T2) compared to that several days before surgery (T1). In all six verbal suggestions and in one of the three non-verbal suggestions, i.e., in seven out of nine tests, muscular performance was lower at T2 (Table 7). A possible explanation for the deviant conduct of the two visual triggers is given above. To our knowledge, this is the first time that a time dependency of nocebo effects has been demonstrated, namely, an impact of the time to a medical intervention. In contrast to healthy volunteers in experimental placebo research, in the clinical situation, patients experience a change of the situation with time. Furthermore, the observed increase in the nocebo effect with the date of surgery approaching is connected to the increase in state anxiety level. Thus, distance to treatment and anxiety are to be considered part of the context sensitivity of nocebo effects. At both preoperative time points, patients showed state anxiety scores exceeding 40, which are considered clinically relevant (Knight et al., 1983; Addolorato et al., 1999). Between days before surgery and the last evening, state anxiety levels increased significantly by 15%, i.e., STAI-S at T2 was 114.8% of the T1 value. Moreover, this increase in anxiety correlated with an increased extent of the induced nocebo effect (Figure 4). Preoperative anxiety is well known (Millar et al., 1995), and an increase during the hospital stay may be expected. Furthermore, a negative correlation of placebo effects and positive correlation of nocebo effects with anxiety level have been described (Corsi and Colloca, 2017). Surprisingly, this study produced evidence that nocebo effects in the clinical context are time sensitive and increase with the extent of an increase in anxiety. While age and gender had no significant effect on the preoperative level of anxiety, both factors proved to be significant independent predictors of the increase in anxiety with the surgical appointment coming closer. While statistically significant but low in extent, the correlation between anxiety level or increase in anxiety, respectively, and reduction in muscle strength by negative suggestions is of clinical relevance. It highlights the time close to a medical intervention as critical with regard to nocebo effects. The results identify young women especially prone to the negative influences of the clinical environment. Fortunately, they respond to alternative formulations to the same extent and thus also can be protected from negative suggestions and nocebo effects. Since pain is a confounder of motor performance (Tucker and Hodges, 2009), we had excluded patients with pain from the study.

Finally, in this study, suggestibility as tested with the HGSHS had an impact on the extent of the nocebo effect (Figure 5). While this correlation is not always observed in placebo research, it may reflect the inclusion of a number of suggestions tested where factors other than conditioning and expectation play a role (Corsi and Colloca, 2017; Hansen and Zech, 2019). The very low regression coefficient confirms the observation that suggestibility is not a major determinant in clinical situations (Montgomery et al., 2011), and that suggestions have impact on all patients, not merely on highly suggestible subjects. A smaller but significant effect of sex and age was observed in this study. While the role of sex on placebo and nocebo effects is being debated (Enck and Klosterhalfen, 2019), we found females more prone to react to negative suggestions both directly and indirectly by increasing in anxiety with the surgery date coming closer. Placebo effects seem to be stronger in children but seem not to differ with age in adults (Wrobel et al., 2016). We found more pronounced nocebo effects in younger patients again both directly and indirectly *via* a higher rate of preoperative increase in anxiety. In conclusion, our results confirm the influence of psychological factors on nocebo responses apart from conditioning and expectation (Corsi and Colloca, 2017). The observed small correlations are a strong argument for a high number of contributing factors.

### Limitations of the Study

A limitation of the study is the incomplete randomization, i.e., the sequence of suggestions was randomized for each patient but adhered to an alternation of negative and positive suggestions. This was because known cumulation effects were to be avoided.

The wide range for T1 (3–10 days before surgery, with two outliers at day 25) could have influenced the test results. Anxiety is a possible factor affecting nocebo effects and is expected to increase with the date of surgery approaching, where the exact course of preoperative anxiety increase remains to be evaluated. However, the strongest increase can be assumed close to the date of surgery, i.e., between day 3 and the evening before surgery, while the difference in anxiety level between days 3 and 9 should be rather low.

The mechanism of the observed effects after negative suggestions remains unclear at both the physiological and psychological level. From a psychological point of view, language-induced motor activity, arousal and affirmation effects, modulation of motor cortex or cortico-spinal excitability, and many more may play a role (Li et al., 2004; Pulvermuller et al., 2005). Considering physiological mechanisms, many are proposed according to the many fields of research like ethology, behavioral and communication research, psychosomatics, hypnosis, and placebo research. Even the latter describes various factors possibly involved like hormones, immune mediators, endogenous opioids, dopamine and other neurotransmitters, and local changes in brain metabolism, microcirculation, and neural functions (Benedetti et al., 2003; Finniss et al., 2010; Benedetti and Amanzio, 2013). Different mechanisms have been described for expectation- or conditioning-induced placebo effects (Amanzio and Benedetti, 1999), like different neurotransmitter involvement (Scott et al., 2008), and the activation of different brain areas (Freeman et al., 2015). A number of excellent and basic studies have evaluated placebo and nocebo effects on motor performance and possible mechanisms (Carlino et al., 2014; Fiorio, 2018; Corsi et al., 2019). In the present study, a motor performance was only used as a marker to assess and quantitate negative or positive effects of clinical suggestions. It is noteworthy that in contrast to the mentioned studies, the tested suggestions here did not contain or relate to words like muscular, power, strength, motion, fatigue and activation, or motor imagery. And still they had profound effects on maximal arm muscle strength.

Similarly, various and different mechanisms are discussed for the effects of suggestions in hypnosis (Barber, 1965; Faymonville et al., 2000; De Benedittis, 2015; Jensen et al., 2015).

It remains unclear, and various factors have to be considered to explain, why the positively formulated alternative suggestions (version B) in most cases did not lead to an increase in muscular strength, as would be expected from their intended placebo effects. This may be due to the fact that although using positive formulations, the surrounding and situation remain a clinical one and thus cannot be positive indeed. Another possible interpretation of the results is a lack of nocebo effects in version B instead of a failed placebo effect. Altogether, this confirms our approach not to label version A of the tested suggestions from the beginning (in section “Materials and Methods,” Tables, and Figures) as “nocebo” and version B as “placebo” to leave these categories for description of the results, not of the study object.

### Clinical Implications

The clinical relevance of this study results from three aspects. First, the reported impairment of muscular strength in surgical patients by common suggestions in medical situations is highly disadvantageous for postoperative mobilization, patient’s safety, and respiration. Especially nurses and physiotherapists may be alarmed by this side effect of careless communication and stimulated to join efforts for positive communication. Second, the tested suggestions were not designed for experiments but rather were taken from everyday clinical practice in common and frequent medical situations. Version B of the tested suggestions can be taken as examples to avoid these negative impacts. Third, the results reveal the opportunity for evidence-guided improvement of therapist–patient communication. Doctors, nurses, and other health care providers, all can benefit from such an approach that beyond personal impressions and subjective valuation provides objective, quantitative, and verifiable data on nocebo effects and its prevention.

The described method of using a single outcome parameter, namely, maximal arm muscle strength, for tests of different verbal and non-verbal suggestions facilitates a direct comparison of effects, development and validation of alternative formulations, and study of compound suggestions. Only with a common outcome parameter for different suggestions can the comprehensive sum effect of all be evaluated. Thus, the described test system could be used for complex interactions of suggestions common in medical situations. Moreover, the tested and observed muscular weakening could be a marker for a more general “weakening effect” of nocebo suggestions and a common, clinically relevant “weakening” of patients in the medical setting (Hansen and Zech, 2019; Zech et al., 2019). It may correspond to and reflect weakening of complex basic physiological functions like homeostasis, recovery, or immune surveillance that are not easy or fast to measure and quantify. Therefore, this parameter could possibly address functional aspects more relevant for holistic medicine than specific tested symptoms and individual test variables. Our results show that the word “pain” or “nauseous” besides provoking and intensifying pain or nausea, respectively, can reduce muscle strength. We hypothesize a general “weakening” of patients by such nocebo effects.

## Conclusion

Nocebo effects are even stronger in the clinical situation of patients than in healthy volunteers in experimental settings. In the medical surrounding, much of the common everyday communication as well as many signals actually comprise negative suggestions that can be identified and quantitated by measuring changes in a uniform physiological function like maximal muscle strength. The latter provides fast and reproducible results for comparison of individual persons, of groups, and of different suggestions originating from the medical environment. Furthermore, the test system can be used to develop and verify better alternatives that avoid negative effects on patients’ health and treatment. This provides a means for improvement of doctor–patient communication in clinical practice.

## Data Availability Statement

The raw data supporting the conclusions of this article will be made available by the authors, without undue reservation.

## Ethics Statement

The studies involving human participants were reviewed and approved by Ethikkommission an der Universität Regensburg, # 13-101-0030, ethikkommission@ukr.de. The patients/participants provided their written informed consent to participate in this study.

## Author Contributions

EH contributed to the study plan and design, supervision, literature search, data analysis, preparation of figures, tables, manuscript, and correction of the manuscript. NZ and MSe contributed to the study design, application for ethic committee approval, literature search, participant recruitment, data collection and analysis, and preparation of the manuscript. MSc contributed to the participant recruitment and data collection and analysis. FZ contributed to the statistical analysis and preparation of figures. TS contributed to the literature search and preparation of the manuscript. All authors contributed to the article and approved the submitted version.

## Conflict of Interest

The authors declare that the research was conducted in the absence of any commercial or financial relationships that could be construed as a potential conflict of interest.

## Abbreviations

ASA score: physical status classification system of the American Society of Anesthesiologists
HGSHS: Harvard Group Scale of Hypnotic Susceptibility
NRS: Numeric Rating Scale (of pain)
OR: operating room
STAI-S: State-Trait Anxiety Inventory.
